# Caregiving burden among caregivers of people with myasthenia gravis

**DOI:** 10.1186/s13023-025-03842-w

**Published:** 2025-06-19

**Authors:** Sarah Dewilde, Nafthali H. Tollenaar, Pierre Boulanger, Annie Archer, Raquel Pardo, Elena Cortés-Vicente, Renato Mantegazza, Fiammetta Vanoli, Sophie Lehnerer, Marc Pawlitzki, Malgorzata Heinrich, Femke De Ruyck, Glenn Phillips, Sandra Paci

**Affiliations:** 1 Services in Health Economics (SHE), Boulevard Lambermont 418, 1030 Brussels, Belgium; 2 Association des Myasthéniques Isolés et Solidaires (AMIS), La Chapelle-en-Serval, France; 3 Myasthenia patient group, AFM-Telethon, Évry-Courcouronnes, France; 4 Asociación Miastenia de España (AMES), Valencia, Spain; 5 Hospital de la Santa Creu i Sant Pau, Barcelona, Spain; 6 Fondazione IRCCS, Istituto Nazionale Neurologico Carlo Besta, Milan, Italy; 7 Associazione Italiana Miastenia e Malattie Immunodegenerative, Milan, Italy; 8 Department of Human Neurosciences, Sapienza University of Rome, Rome, Italy; 9 Charité –Universitätsmedizin Berlin, corporate member of Freie Universität Berlin and Humboldt-Universität zu Berlin, Department of Neurology with Experimental Neurology, Neuroscience Clinical Research Center, Berlin, Germany; 10 Digital Health Center, Berlin Institute of Health at Charité – Universitätsmedizin Berlin, Berlin, Germany; 11 Department of Neurology, Medical Faculty and University Hospital Düsseldorf, Heinrich Heine University Düsseldorf, Düsseldorf, Germany; 12 Vitaccess, Oxford, UK; 13 Argenx BV, Ghent, Belgium

**Keywords:** Caregiver burden, Myasthenia gravis, ZBI-22, Informal caregiver, Quality of life

## Abstract

**Introduction/aims:**

Informal caregivers play an important role in the lives of people with Myasthenia gravis (MG). This study aims to assess the caregiver burden (CB) experienced by caregivers of MG patients.

**Methods:**

A cross-sectional study design collected patient and caregiver data in Germany, Italy, Spain, the UK, and France. The Zarit Burden Interview (ZBI-22), EQ-5D-5L and bolt-on questions, and PROMIS Global Health-10 were used to measure CB and overall health-related quality of life (HRQoL).

**Results:**

Caregivers (N = 69) reported a mean ZBI-22 score of 24.3, with 40.6% reporting no burden, 47.8% mild-to-moderate burden, 8.7% moderate-to-severe burden, and 2.9% severe burden. The most impacted ZBI-22 dimensions were “losing control over one’s life”, “financial burden” and “relationships with relatives”. Based on a generic health-related quality of life scale (EQ-5D-5L plus bolt-on questions), the dimensions that were more frequently reported among MG caregivers to cause moderate-to-extreme problems were: tiredness (43%), self-confidence (32%), and anxiety/depression (28%). EQ-5D-5L utilities (values from -1 to 1, reflecting overall HRQoL) were negatively associated with higher ZBI-22 scores (little or no burden: 0.942, mild-to-moderate burden: 0.864, moderate-to-severe burden: 0.783, severe burden: 0.570). Regarding PROMIS-10 items, 48% of caregivers reported often/always being bothered by anxiety, depression, or irritation; 47% reported being not at all or little able to carry out daily activities; and 37% reported having (very) severe fatigue.

**Conclusion:**

Informal caregivers of MG patients experience a substantial burden, impacting their physical, emotional, and financial well-being. Findings highlight the need for tailored interventions to alleviate CB and enhance the caregivers’ overall HRQoL.

## Introduction

Informal caregivers play a vital and often unrecognized role in the lives of many patients. Unlike professional caregivers, informal caregivers provide unpaid care for someone they are related to through family, friendship, or otherwise [1]. They take on diverse responsibilities, ranging from assisting with daily tasks to offering emotional support and managing medical appointments, often resulting in a burden experienced by the caregiver themselves [2]. Caregiver burden has been defined as *“the level of multifaceted strain perceived by the caregiver from caring for a family member and/or loved one over time”* [3]*.* In general, this burden consists of a range of physical, emotional, social, and financial problems, impacting their health-related quality of life (HRQoL), and overall functioning [4]. By recognizing the potential effects of the caregiving burden on caregivers themselves, interventions and support programs can be developed to alleviate these effects and improve caregivers’ well-being.

Myasthenia gravis (MG) is a chronic autoimmune disorder affecting the neuromuscular junction of the skeletal muscles, with a prevalence up to 12.4/100.000 persons. Individuals with MG may experience a various combination of symptoms, including diplopia, ptosis, and problems with swallowing, speaking, breathing, breathing and mobility. They all substantially impact the HRQoL of people suffering from MG and limit their ability to complete activities of daily living independently [5–9]. To cope with these impairments and limitations, many MG patients receive support from an informal caregiver, usually their spouse, family members, or friends [2]. This support is crucial, as individuals without an informal caregiver have been observed to have a lower HRQoL, and more problems with mental health and performing daily activities [10].

The burden experienced by informal caregivers of people suffering from MG has been a subject of research in four previous studies. A study from Germany demonstrated that disease severity was strongly associated with patients’ HRQoL as well as with the caregiver burden [11]. Results from a qualitative study also implied that not only the caregiver but the whole family was impacted by the MG of their relative [2]. Another German study concluded a dependence of patients on their social support networks, and a notable impact on family planning [10]. Finally, a study conducted in northwestern China, highlighted a strong economic strain and disruption of daily activities as most severe aspects of MG-related family burden [12]. Further research is needed to explore the complexities of MG-related caregiver burden across different instruments, settings and countries [2].

Our Caregiver Burden-MG study aims to complement existing evidence by assessing the burden faced by informal caregivers of people with MG, including the impact on their HRQoL, relationship with the patient, emotions, family life, and finances.

## Methods

### Study design and data collection

This cross-sectional study assessed the caregiver burden of MG in five European countries: France, Germany, Italy, Spain, and the UK.

In Germany, Italy, Spain and the UK, pairs of MG patients and their informal caregiver were recruited via the ongoing MyRealWorld-MG study; a digital, observational study conducted among 1859 adults diagnosed with MG from nine countries. The MyRealWorld-MG study’s aim was to offer a comprehensive real-world, long-term assessment of the impact of MG in a large, diverse cohort, from the perspective of those affected by MG. Using a smartphone application, patients entered data on disease characteristics (diagnosis, disease duration, antibody status, received treatments) and reported monthly on their experience living with MG during a 2-year period. Patients were informed about the Caregiver Burden-MG study if they reported receiving regular help from a caregiver and consented to be approached for future research. However, only a proportion of participants met these conditions and found their caregiver willing to participate, resulting in a relatively low sample size. Patients informed their caregiver by providing them with the study website. Both were to provide consent before enrolling in the Caregiver Burden-MG study. Caregiver and patient filled in the online questionnaire and their data were later matched for analysis.

In France, recruitment of patient-caregiver pairs was different due to local policy. Pairs of MG patients and their informal caregivers were recruited through patient advocacy groups. After giving consent, patients and caregivers separately received a paper-based survey, which they sent back after completion. As with the pairs from other countries, reported outcomes were matched for analysis. Data from all five countries were subsequently combined and analyzed.

### Participants

Caregivers had to be adults, and the principal informal caregiving person. Their caregiving burden was evaluated using the Zarit Burden Interview (ZBI-22), and HRQoL was measured with the EuroQol 5-Dimension 5-Level (EQ-5D-5L) and the PROMIS v1.2—Global Health 10 (PROMIS-GH). Adult patients with a diagnosis of MG completed the MG Activities of Daily Living (MG-ADL) instrument and the EQ-5D-5L. Additionally, data on patient and caregiver demographics, their relationship, and the number of caregiving hours were collected.

### Measures

#### Zarit burden interview (ZBI-22)

The Zarit Burden Interview (ZBI-22) is a widely used tool for assessing the subjective burden experienced by caregivers consisting of 22 items [13]. Each item is rated on a 5-point Likert scale ranging from 0 (never) to 4 (nearly always), with a total score ranging from 0 to 88. A higher total ZBI-22 score indicates a higher caregiver burden. A total score of 0–21 was considered as “little or no burden”, 21–40 as “mild-to-moderate burden”, 41–60 as “moderate-to-severe burden”, and 61–88 as “severe burden” [14].

Although the instrument was developed as a unidimensional scale, its questions have been divided into 5 dimensions to report the impact of disease on caregivers in a more concise way: burden in the relationship (6 items), caregiver’s emotional well-being (7 items), social and family life (4 items), finances (1 item), and loss of control over one’s life (4 items) [15].

#### EQ-5D-5L

The EQ-5D-5L is a generic HRQoL measure, consisting of a descriptive system assessing general health on five dimensions: mobility, self-care, usual activities, pain/discomfort and anxiety/depression. Each dimension is described in five severity levels ranging from “having no problems” to “having extreme problems / being unable” to perform the action on that dimension on the day of completion [16]. Six “bolt-on dimensions” were added to the standard EQ-5D dimensions to be able to capture disease-related quality of life impairments in addition to the general quality of life aspects, including sleep [17], tiredness [18], social relationships [19], and self-confidence [19] with the same five response levels as the main descriptive system [20].

Furthermore, the visual analogue scale (VAS) is a thermometer-like vertical scale ranging from 0 (worst imaginable health) to 100 (best imaginable health) on which respondents need to rate their overall health on the day of completion [16]. Responses on the five core dimensions can be combined into the utility value, anchored by a value of 1 for full health and 0 for dead, whereas health conditions perceived as worse than death are denoted by negative values [21].

#### PROMIS v1.2—global health 10

The PROMIS-GH is, like the EQ-5D-5L, a validated, generic HRQoL outcome measure. However, it contains twice as many items (hence often referred to as PROMIS-10), thus providing a more comprehensive assessment of general health than the EQ-5D-5L. The PROMIS-GH instrument comprises two subscales: Global Physical Health (GPH) and Global Mental Health (GMH), each consisting of four items [22]. Items are scored on a 5-point Likert scale, with the exception of one item that uses an 11-point scale to rate pain levels. Based on the results, T-scores for GPH and GMH can be calculated using the HealthMeasures Scoring Service [23], with lower values indicating poorer health. Notably, the calculation excludes two items—general health (*Global01*) and social roles (*Global09*).

#### MG-ADL

The MG-ADL is a widely used assessment tool to evaluate the functional abilities of people with MG [24]. It consists of 8 common MG symptoms with each response graded from 0 to 3. Cumulative scores range from 0 to 24, with a higher score indicating greater limitations. We used the MG-ADL total score to categorize patients as having mild (0–4), moderate (5–9), or severe (> = 10) MG. This categorization was used in our previous MG-related publications, follows the advice of neurologists, and mirrors the inclusion criteria from clinical trials where a score of 5 or higher was used to classify patients as moderate to severe [8, 25, 26].

### Statistical analysis

Sample characteristics were presented using descriptive analysis. Continuous variables were described using the mean, median, standard deviation, the inter-quartile range and the 5th and 95th percentiles, whilst categorical variables were described using proportions. All instruments were scored according to the scoring manual; missing data were not imputed as there were no missing data in the dataset.

The results from this survey were compared with general population norms or with published data. The T-test was used to examine differences between our study results and external data when data were normally distributed, whereas the Wilcoxon Rank Sum test was used for non-normally distributed variables, if patient-level data was available. The Shapiro–Wilk W test was used to assess the normality of the distribution [27]. In case where a categorical variable was compared, a Chi-Square test was used to test for the overall difference in the distribution.

General Population Norms data for the EQ-5D was available through POPUP, an observational digital study reporting international norms for the EQ-5D-5L in 8 countries [28, 29]. EQ-5D-5L utility values for caregivers from all countries were calculated using the French value set, as France had the largest contribution to the sample size. These utility values were compared with values from the general population, based on data from the same countries and matched by age and gender, and using the same value set, which allows for a direct comparison [30]. The ZBI-22 scores were compared using summary data from published literature with caregiving burden scores from other disease areas; no reported ZBI-22 score norms were found for the general population. For the PROMIS-Global Health, we calculated T-scores for the physical (GPH) and mental (GMH) subscales and compared them with standardized US population norms of mean (SD) T-score 50 (10), and in the discussion section we refer to norms for the Dutch [31] and Hungarian [32] general populations to interpret our findings.

## Results

### Study population

A total of 69 pairs – consisting of an adult suffering from MG and their informal caregiver – were included in this study (Table 1). MG patients had a mean age of 50.6 years and were predominantly female. Caregivers had a mean age of 53.7 years and were more likely to be male. Based on self-reported total MG-ADL scores, the patients participating in the Caregiver Burden-MG study suffered from mild (41%), moderate (36%) or severe MG (23%). These were similar proportions as in the overall patient population from MG cohort MyRealWorld-MG (41.7% mild, 39.6% moderate and 18.7% severe MG, *p* = 0.52) [8].

**Table 1:** Demographic characteristics of the respondents

Characteristics		Caregivers (N = 69)	Patients (N = 69)
Age categories	18–40 years old	19%	30%
	40–60 years old	40%	44%
	60 + years old	40%	25%
Age, years	Mean	53.7	50.6
	(Median, SD)	(54, 14.1)	(51, 14.6)
Gender	% Female	39%	75%
	% Male	61%	25%
Countries	Germany	11%	
	Spain	14%	
	France	40%	
	Italy	34%	
	UK	2%	
MG-ADL total score of person diagnosed with MG	Mild: 0–4	41%	
	Moderate: 5–9	36%	
	Severe: 10 and over	23%	
Disease duration, years	Mean	13.6	
	(Median, SD)	(9, 12.7)	

MG, myasthenia gravis; MG-ADL, Myasthenia gravis-activities of daily living scale; SD, standard deviation

The vast majority (84%) of caregivers were the spouse/life partner of the individual with MG they provided care for, and 90% of pairs lived together (Table 2). The mean (SD) daily caregiving hours were 5.1 (6.5), with 20% of caregivers providing care for 15–49 h/week and 30% for 50 + hours/week. Furthermore, a quarter of caregivers reported they had to reduce working hours due to their responsibilities for the individual with MG they provided care for.

**Table 2:** Details of the caregiving situation and intensity, as reported by caregivers

Caregiving specifics		%
Relationship to person diagnosed with MG	Spouse/life partner	84%
	Parent	4%
	Child	3%
	Brother/sister	4%
	Friend	1%
	Other	3%
Living together with the person affected by MG?	Yes	90%
How long have you been caring for the person affected by MG?	Years, Mean (Median, SD)	9.9 (6, 10.2)
Other caregivers for same MG patient (e.g., family members, paid caregivers)?	Yes	37%
Other dependents (e.g. children, parents)?	Yes	38%
Hours per day caregiving	Hours, Mean (Median, SD)	5.1 (3, 6.5)
Hours per week caregiving	0–7	26%
	8–14	24%
	15–49	20%
	50 +	30%
Impact on ability to work, or reduction in working hours?	Yes	25%
Last MG-related hospitalization of the person diagnosed with MG	< 2 years ago	43%
	> 2 years ago	45%
	Never been hospitalized due to MG	12%

MG, myasthenia gravis, MG-ADL, Myasthenia Gravis-Activities of Daily living scale, SD, standard deviation

### Caregiver burden (ZBI-22)

The mean (SD) ZBI-22 score for all caregivers was 24.3 (15.0). One in ten caregivers experienced a moderate-to-severe or severe burden, half of caregivers reported a mild-to-moderate burden, and the rest had little or no burden.

#### ZBI-22 dimensions

The proportional burden (expressed as a range between 0% = no burden, to 100% = maximal burden) was largest for the dimension “Losing control over ones’ life”, closely followed by the dimension “financial burden” and the dimension “impact of MG on the relationship with their relatives”. The average burden on “emotional well-being” and “social and family life” was similar and markedly lower compared to the other dimensions.

#### ZBI-22 items

Items that caregivers indicated they worried about *frequently* or *nearly always* covered a wide array of feelings, and multiple aspect of life (Table 3). Common worries related to feeling afraid about what the future holds for their relative and the uncertainty about what to do about them. Also, many caregivers felt they did not have enough time and money to manage their caregiving duties alongside their other commitments and expenses. Caregivers often felt like the individual with MG depended on them, or even expected them to provide care as if they were the only one they could count on. One-third of caregivers felt *sometimes* to *nearly always* that they have lost control of their life because of their caring responsibilities, and/or that their health has suffered because of this. Still, the vast majority of caregivers felt like they should be doing more—and better. On the other hand, the majority of caregivers never felt embarrassed about the care recipient’s behavior, never felt uncomfortable to host friends due to the relative’s condition, and never wished they could leave the care of their relative to someone else.

**Table 3:** Distribution of caregivers on the Zarit Burden Interview questionnaire

Zarit Burden Interview	Caregivers (N = 69)				
ZBI Items	Never	Rarely	Sometimes	Frequently	Nearly Always
1. Do you feel that your relative asks for more help than he/she needs?	54%	19%	20%	3%	4%
2. Do you feel that because of the time you spend with your relative that you don’t have enough time for yourself?	37%	34%	19%	3%	7%
3. Do you feel stressed between caring for your relative and trying to meet other responsibilities for your family or work?	26%	16%	41%	9%	9%
4. Do you feel embarrassed over your relative’s behavior?	64%	16%	17%	1%	1%
5. Do you feel angry when you are around your relative?	48%	25%	22%	3%	3%
6. Do you feel that your relative currently affects our relationships with other family members or friends in a negative way?	57%	22%	14%	6%	1%
7. Are you afraid what the future holds for your relative?	9%	13%	35%	28%	16%
8. Do you feel your relative is dependent on you?	23%	14%	26%	23%	13%
9. Do you feel strained when you are around your relative?	57%	19%	22%	3%	0%
10. Do you feel your health has suffered because of your involvement with your relative?	54%	14%	20%	7%	4%
11. Do you feel that you don’t have as much privacy as you would like because of your relative?	56%	28%	10%	3%	3%
12. Do you feel that your social life has suffered because you are caring for your relative?	48%	17%	25%	7%	3%
13. Do you feel uncomfortable about having friends over because of your relative?	77%	10%	10%	1%	1%
14. Do you feel that your relative seems to expect you to take care of him/her as if you were the only one he/she could depend on?	43%	10%	16%	16%	14%
15. Do you feel that you don’t have enough money to take care of your relative in addition to the rest of your expenses?	42%	16%	16%	19%	7%
16. Do you feel that you will be unable to take care of your relative much longer?	57%	20%	14%	6%	3%
17. Do you feel you have lost control of your life since your relative’s illness?	49%	17%	20%	10%	3%
18. Do you wish you could leave the care of your relative to someone else?	65%	16%	14%	3%	1%
19. Do you feel uncertain about what to do about your relative?	36%	19%	28%	14%	3%
20. Do you feel you should be doing more for your relative?	22%	25%	33%	13%	7%
21. Do you feel you could do a better job in caring for your relative?	25%	25%	33%	10%	7%
22. Overall, how burdened do you feel in caring for your relative?	43%	33%	10%	9%	4%
**ZBI Categories**					
0–21 little or no burden	41%				
21–40 mild to moderate burden	48%				
41–60 moderate to severe burden	9%				
61–88 severe burden	3%				

Variable	Mean	SD	5th Pctl	Lower quartile	Median	Upper quartile	95th Pctl
Total ZBI score	24.3	15.0	5.0	14.0	23.0	29.0	53.0
Burden in the relationship	30%	19%	4%	17%	25%	42%	63%
Emotional wellbeing	24%	20%	4%	11%	21%	29%	64%
Social and family life	23%	20%	0%	13%	19%	31%	63%
Finances	33%	35%	0%	0%	25%	75%	100%
Loss of control over ones’ life	34%	20%	6%	19%	31%	50%	69%

Abbreviations: Pctl, Percentile, SD, standard deviation, ZBI, Zarit Burden Interview

### EQ-5D-5L

#### Moderate-to-extreme problems per dimension

Caregivers struggled most with anxiety/depression, with 28% reporting moderate-to-extreme problems (Table 4), significantly more than in the general population (17%, *p* = 0.014). Moderate-to-extreme problems with usual activities were also reported significantly more by caregivers compared to the general population (12% vs. 2%, *p* < 0.001). By contrast, moderate-to-severe problems with pain/discomfort, mobility, and self-care (18%, 12%, and 4% respectively) did not significantly differ from the general population (*p* = 0.152 to *p* = 0.618). Mean EQ VAS values were similar in caregivers (72.8) and the general population (75.6, *p* = 0.28). The mean caregiver utility value was 0.879, a decrement of 0.047 (*p* = 0.0046) compared to the utility value of 0.926 for members of the general population (matched by country, age and gender).

**Table 4:** Distribution of respondents across the levels per domain of the EQ-5D-5L

Domains	Caregivers	General population^a^
	(N = 69)	(N = 9000)
*Mobility*		
I have no problems walking	76%	79%
I have slight problems walking	12%	13%
I have moderate problems walking	7%	5%
I have severe problems walking	3%	2%
I am unable to walk	1%	0%
*Self-care*		
I have no problems washing or dressing myself	90%	91%
I have slight problems washing or dressing myself	6%	6%
I have moderate problems washing or dressing myself	3%	2%
I have severe problems washing or dressing myself	1%	1%
I am unable to wash or dress myself	0%	0%
*Usual Activities*		
I have no problems doing my usual activities	66%	80%
I have slight problems doing my usual activities	22%	13%
I have moderate problems doing my usual activities	6%	5%
I have severe problems doing my usual activities	4%	2%
I am unable to do my usual activities	1%	0%
*Pain / Discomfort*		
I have no pain or discomfort	31%	49%
I have slight pain or discomfort	51%	36%
I have moderate pain or discomfort	10%	12%
I have severe pain or discomfort	6%	3%
I have extreme pain or discomfort	1%	1%
*Anxiety / Depression*		
I am not anxious or depressed	41%	54%
I am slightly anxious or depressed	31%	29%
I am moderately anxious or depressed	16%	12%
I am severely anxious or depressed	10%	4%
I am extremely anxious or depressed	1%	1%
*Total level sum score*		
Mean	8.1	7.1
SD	3.4	2.7
5th Pctl	5.0	5.0
Lower quartile (Q1)	6.0	5.0
Median	7.0	6.0
Upper quartile (Q3)	9.0	8.0
95th Pctl	15.0	13.0
*Utility*		
Mean	0.879	0.926
SD	0.206	0.143
5th Pctl	0.578	0.659
Lower Quartile (Q1)	0.898	0.930
Median	0.956	0.978
Upper Quartile (Q3)	0.980	1.000
95th Pctl	1.000	1.000
*VAS*		
Mean	72.8	75.6
SD	18.8	17.5
5th Pctl	40.0	40.0
Lower quartile (Q1)	60.0	69.0
Median	74.0	80.0
Upper quartile (Q3)	90.0	90.0
95th Pctl	96.0	97.0

Pctl, Percentile, SD, standard deviation, VAS, visual analogue scale. ^a^ Data from the general population study POPUP

#### Moderate-to-extreme problems in bolt-on questions

Out of the 4 bolt-on dimensions to the EQ-5D-5L, tiredness and self-confidence emerged as the ones caregivers struggled with the most (Table 5). Compared to the general population, more than double the proportion of caregivers reported having moderate-to-extreme problems with tiredness (43% vs. 21%, *p* < 0.001) and with self-confidence (32% vs. 14%, *p* = 0.007). By contrast, moderate-to-extreme problems with sleeping (21% vs. 22%, *p* = 0.763) and maintaining social relationships (15% vs. 21%, *p* = 0.187) were reported less by caregivers compared to the general population.

**Table 5:** Distribution of respondents on the EQ-5D-5L bolt-on questions

Domains	Caregivers	General population^a^
	(N = 69)	(N = 9000)
*Tiredness*		
I am not tired	22%	47%
I am slightly tired	35%	32%
I am moderately tired	26%	15%
I am severely tired	13%	5%
I am extremely tired	3%	1%
*Sleep*		
I have no problems sleeping	41%	38%
I have slight problems sleeping	38%	40%
I have moderate problems sleeping	12%	16%
I have severe problems sleeping	6%	5%
I have extreme problems sleeping	3%	1%
*Self-confidence*		
I have no problems with self-confidence	52%	66%
I have slight problems with self-confidence	16%	21%
I have moderate problems with self-confidence	16%	9%
I have severe problems with self-confidence	16%	4%
I have extreme problems with self-confidence	0%	1%
*Social relationships*		
I have no problems with social relationships	66%	51%
I have slight problems with social relationships	19%	28%
I have moderate problems with social relationships	10%	13%
I have severe problems with social relationships	3%	6%
I have extreme problems with social relationships	1%	2%

^a^Data from the general population study POPUP

### Association ZBI-22 and EQ-5D-5L utility values

**Fig. 1:**
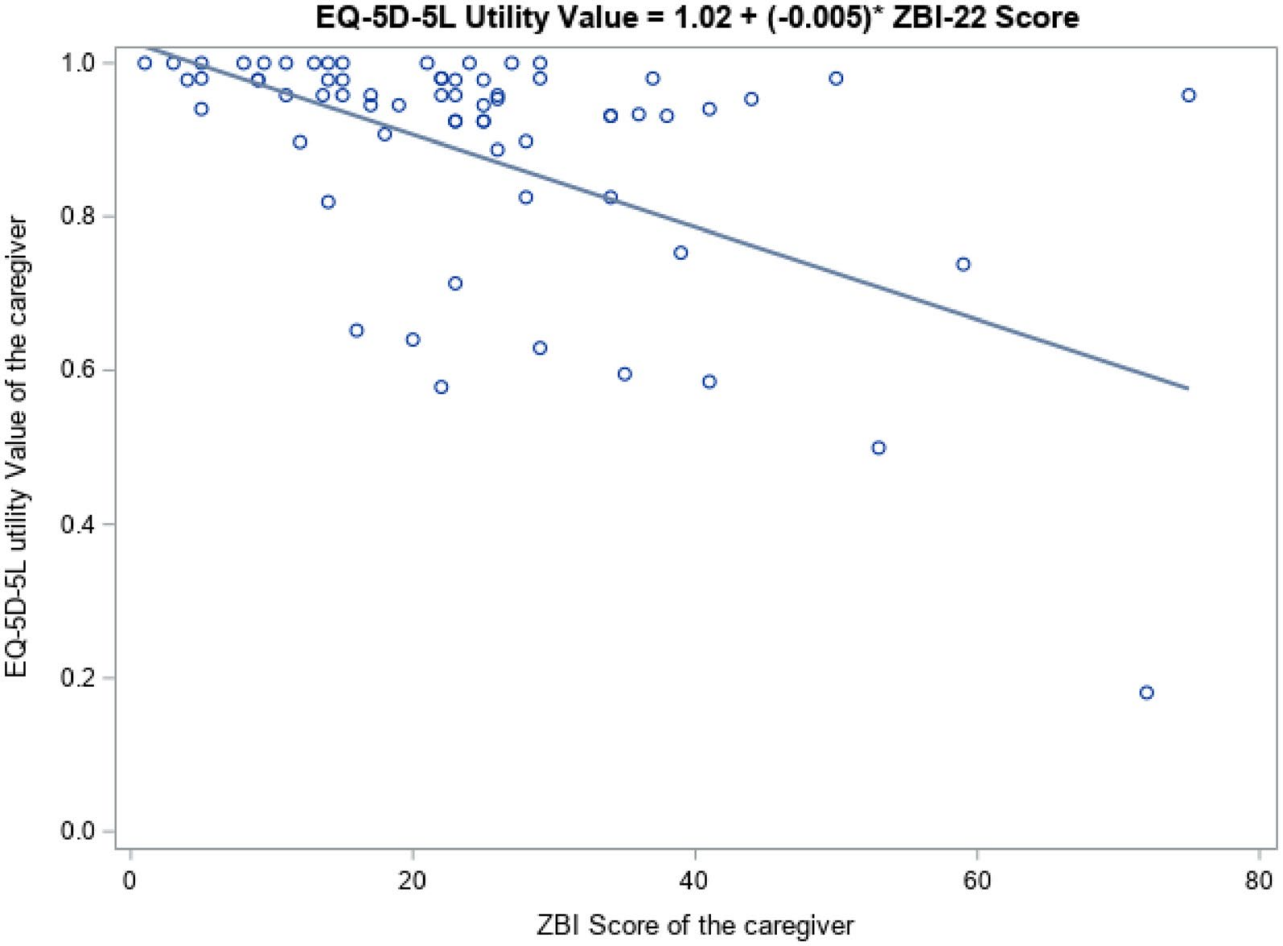
**EQ-5D-5L utility values by total ZBI score**

EQ-5D-5L utility values decreased with ZBI-22 scores (Table 6, *p* < 0.0001), ranging from 0.942 in caregivers with little or no burden (0–21) to 0.570 in caregivers with severe burden (61–88). Figure 1 plots these utility values by the ZBI-22 scores, showing an overall downward trend. This means that the caregiver’s HRQoL declines when the caregiver burden increases. However, the scattered distribution of bubbles across the plot highlights that the variability in overall HRQoL increases as the burden experienced by the caregiver becomes heavier.

**Table 6:** Utility values by ZBI score

ZBIcat	Mean	SD	5th Pctl	Lower Quartile	Median	Upper Quartile	95th Pctl
**0–20** **Little or no burden**	0.942	0.095	0.652	0.945	0.978	1	1
**21–40** **Mild to moderate burden**	0.864	0.233	0.578	0.887	0.931	0.978	1
**41–60** **Moderate to severe burden**	0.783	0.207	0.499	0.585	0.839	0.953	0.98
**61–88** **Severe burden**	0.57	0.549	0.181	0.181	0.57	0.958	0.958

EQ-5D-5L, EuroQoL 5-Dimension 5-Level, SD, standard deviation, ZBI, Zarit Burden Interview Pctl, Percentile

### PROMIS global health

Mean Global Physical Health and Global Mental Health T-scores of caregivers for individuals with MG were 40.6 (SD 8.5) and 42.7 (SD 9.8), which was considerably lower than the general population (both *p* < 0.0001). Emotional problems were prevalent in a large proportion of caregivers, with almost half the caregivers reporting that they are always (27%) or often (21%) bothered by anxiety, depression, or irritation (Table 7). Approximately half the caregivers reported not being able to carry out everyday physical activities at all (34%) or only a little (13%). Additionally, about four out of ten caregivers reported having poor (12%) or fair (29%) mental health, and very severe (16%) or severe (21%) fatigue in the past 7 days. Poor or fair physical health was experienced by 7% and 13%, respectively. A quarter of all caregivers described their general health and quality of life as poor (6%) or fair (18%).

**Table 7:** Distribution of caregivers on the PROMIS Global Health—10 questionnaire

PROMIS global health—10		Distribution of responses over the response categories (N = 68)				
Item		Poor (1)	Fair (2)	Good (3)	Very good (4)	Excellent (5)
Global01	In general, would you say your health is:	6%	18%	35%	31%	10%
Global02	In general, would you say your quality of life is:	6%	18%	43%	28%	6%
Global03	In general, how would you rate your physical health:	7%	13%	41%	28%	10%
Global04	In general, how would you rate your mental health, including your mood and your ability to think:	12%	29%	31%	18%	10%
Global05	In general, how would you rate your satisfaction with your social activities and relationships:	7%	19%	44%	22%	7%
Global09r	In general, please rate how well you carry out your usual activities and roles:	6%	26%	38%	26%	3%
Global06	To what extent are you able to carry out your everyday physical activities such as walking, climbing stairs, carrying groceries or moving a chair?	Not at all (1)	A little (2)	Moderately (3)	Mostly (4)	Completely (5)
		34%	13%	15%	18%	21%
		Always (1)	Often (2)	Sometimes (3)	Rarely (4)	Never (5)
Global10r	In the past 7 days, how often have you been bothered by emotional problems such as feeling anxious, depressed or irritable?	27%	21%	24%	22%	6%
		Very severe (1)	Severe (2)	Moderate (3)	Mild (4)	None (5)
Global08r	In the past 7 days, how would you rate your fatigue on average?	16%	21%	36%	25%	1%
		Mean	SD	Q1	Median	Q3
Global07r	In the past 7 days, how would you rate your pain on average? Worst pain imaginable = 10 9 8 7 6 5 4 3 2 1 0 = No Pain	3.3	2.5	1.0	3.0	6.0

Subscales	Mean	SD	5th Pctl	Lower Quartile	Median	Upper Quartile	95th Pctl
Mental Health—T Score (General population norm = 50)	42.7	9.8	24.1	36.4	43.3	49.3	59.4
Physical Health—T Score (General population norm = 50)	40.6	8.5	24.0	35.3	40.1	46.6	54.4

SD, standard deviation, Pctl, Percentile

### Comparison of the caregiving burden between countries

The overall HRQoL and burden of caregivers was similar between countries as measured by the EQ-5D-5L (*p* = 0.98), the ZBI-22 (*p* = 0.64), and the PROMIS-GMH scores (*p* = 0.42) (Table 8). However, the physical health of caregivers based on the PROMIS-GPH was substantially different between countries (*p* < 0.0001); caregivers in France reported notably higher (better) scores than caregivers in Italy, Germany, Spain or the UK.

**Table 8:** HRQoL and Burden scores by country

	France				
	Mean	SD	Median	IQR	5th Pctl, 95th Pctl
EQ-5D-5L utility	0.879	0.251	0.958	(0.897; 0.98)	(0.713; 1)
ZBI total score	25.6	13.6	24.5	(14; 36)	(9; 50)
PROMIS10 mental health	44.2	8.6	44.8	(39; 50)	(31; 59.4)
PROMIS10 physical health	46.3	7.9	46.3	(42; 54)	(35.2; 57.8)
	Italy				
	Mean	SD	Median	IQR	5th Pctl, 95th Pctl
EQ-5D-5L utility	0.885	0.192	0.945	(0.924; 0.98)	(0.578; 1)
ZBI total score	24.2	18.5	22.0	(14; 26)	(4; 72)
PROMIS10 mental health	43.0	9.6	42.9	(38; 47)	(30.3; 55.6)
PROMIS10 physical health	38.2	6.2	38.0	(35; 42)	(27.3; 47.2)
	**Germany, Spain, UK**				
	Mean	SD	Median	IQR	5th Pctl, 95th Pctl
EQ-5D-5L utility	0.874	0.168	0.945	(0.825; 1)	(0.585; 1)
ZBI total score	22.7	13.0	23.0	(14; 29)	(5; 41)
PROMIS10 mental health	40.4	11.4	37.8	(34; 50)	(21.3; 56.8)
PROMIS10 physical health	36.1	7.4	35.4	(31; 42)	(24; 49.2)

EQ-5D-5L, EuroQoL 5-Dimension 5-Level, IQR, Inter Quartile Range, SD, standard deviation, ZBI, Zarit Burden Interview, Pctl, Percentile

## Discussion

This study revealed a considerable burden experienced by informal caregivers of MG patients using the ZBI-22, EQ-5D-5L, and PROMIS-GH instruments. Caregivers reported a range of physical, emotional, social, and financial consequences of their caregiving responsibilities. The demands of caregiving were found to be large, with a notable proportion of caregivers experiencing stress and feeling overwhelmed by their responsibilities. Financial strain was also evident, with caregivers often feeling inadequate to cover caregiving costs alongside their regular expenses. The burden experienced by the caregivers was similar in all countries, though the impact on their physical health may be different. Many factors could be at the basis for this, including different patient characteristics (age, gender), different disease severity, local availability of additional sources of care and access to medical help, and the characteristics of the caregivers themselves (age, gender, own physical condition).

### Comparison of the ZBI-22 caregiver burden score to other (neurological) diseases

Marbin et al. (2022) aimed to show the impact of mental health issues and (self-perceived) MG severity on the HRQoL of caregivers [11]. Caregivers had a mean Burden Scale for Family Caregivers (BSFC) score of 3 (range 0–30), which is low (25th percentile) according to a validation study of the scale, indicating a relatively low caregiving burden in that study [33]. A possible explanation is the smaller proportion of patients with severe MG in the Marbin study compared to this analysis (12.8% according to the MG-QoL15 scores in Marbin vs. 23% according to the MG-ADL category in this study), since BSFC scores of caregivers were strongly associated with disease severity and the patients’ HRQoL.

The caregiver burden measured with the ZBI-22, was found to be dependent on the patient’s underlying condition, ranging from 34.1 in dementia/cognitive impairment, 32.6 in mental illnes*s*, and 32.5 in Alzheimer’s, 27.0 in physically disabled people, 26 in Amyotrophic Lateral Sclerosis (ALS) [34] and 24.6 in elderly/dependent persons [35]. Based on this range of ZBI-22 scores, the burden of caregivers of people suffering from MG is best comparable with caregivers for physically disabled people and elderly/dependent persons. Additionally, substantial differences in mean ZBI-22 scores were found according to the patient’s disease severity in a study conducted among Alzheimer’s disease patients (ZBI-22 scores for mild 25.8, moderate 35.6 and severe 42.6 disease). Marbin et al. found a similar effect of disease severity on the caregiver burden in MG, and it would be valuable to explore this association in further research.

### Comparison of the EQ-5D utility values to the general population

The heightened anxiety and depression levels reported among caregivers may be attributed to the unpredictable, variable, fluctuating, and often invisible nature of myasthenia gravis. Problems among caregivers with tiredness and self-confidence may be linked to the considerable physical and emotional demands associated with caregiving, often extending beyond prior capacities and disrupting existing expectations [36]. We are unable to provide a logical explanation why moderate-to-extreme problems with maintaining social relationships were more commonly reported by the general population.

### Comparison of the EQ-5D-5L utility values to other caregivers

Although the neurodegenerative disorder metachromatic leukodystrophy (MLD) and MG are distinct disorders with different underlying causes, both result in muscle weakness, respiratory and swallowing difficulties, and vision impairment. A recent international study of caregiver burden in MLD (n = 34) showed a markedly lower proportion of MLD caregivers reporting any problems with EQ-5D-5L mobility (9% vs. 24%, *p* = 0.072) and self-care (3% vs. 10%, *p* = 0.193), compared to our MG caregivers [37], however samples were small which makes it difficult to validate differences. In contrast, the proportion of caregivers experiencing pain or discomfort (65% vs. 69%, *p* = 0.653) was similar to our results, whereas difficulties with performing usual activities (41% vs. 34%, *p* = 0.466), and anxiety and depression (68% vs. 59%, *p* = 0.387) were more frequently reported among MG caregivers than MLD caregivers. Importantly, it should be noted that all caregivers in the MLD study were parents of children with MLD, whereas in our study caregivers are mostly spouses of adult patients, hence the higher age will be a confounding factor in this comparison, The younger age of the MLD caregivers could explain why they reported having less problems with EQ-D-5L mobility and self-care. Both mean EQ-5D-5L utilities (0.845 vs. 0.879) and VAS values (71.2 vs. 72.8) were within the same range for informal caregivers of ALS and MG patients.

### Comparison of the PROMIS-GH scores to the general population

The reported T-scores of caregivers for both Global Physical Health (40.6) and Global Mental Health (42.7) in this study are considerably lower than the average US population norm of mean = 50 (SD = 10) [38]. The difference with the general population is smaller when comparing the T-scores of caregivers for individuals with MG to European population norms instead of the standardized US norms. Where Hungarian population norms [32] (Global Physical Health: 49.0, Global Mental Health: 47.7) are still notably higher compared to T-scores of caregivers (but lower than US population norms), the difference with the Dutch values [31] (Global Physical Health: 45.2, Global Mental Health: 44.7) is smaller but still significant (*p* < 0.0001 and *p* = 0.02).

Overall, the literature reveals the complexity of caregiver burden in various neurological disorders, including MG. Based on the literature in other neurological conditions, the caregiver burden may be influenced by multiple factors, including patient characteristics, caregiver characteristics, disease severity, and the availability of social support. A tandem paper will explore the impact of these factors on the caregiver burden of MG.

### Limitations

While this study offers valuable insights into the burden experienced by informal caregivers of people with MG, there are several limitations that should be acknowledged. Firstly, the sample size of our study is small and all included countries were located in Europe, which limits the generalizability of our findings as well as the ability to compare them to other studies. Secondly, the cross-sectional design does not provide insights into causality or in longitudinal changes in caregiver burden over time, and factors impacting or alleviating the burden were not discussed in this manuscript. However, an in-depth analysis of the impact of patient and caregiver characteristics on the caregiving burden, including disease severity, will be published in a tandem paper. In addition, some variables that might impact the analyses were not captured in our data collection, such as other chronic conditions among MG patients. Lastly, selection bias might have occurred, since a study on caregiver burden might be most interesting to participate in for those who are experiencing a larger burden and seeking support.

## Conclusion

In this small study, we assessed the burden faced by informal caregivers of people with MG. The findings provide insights into the diverse challenges that caregivers encounter, spanning physical, emotional, social, and financial domains. The emotional strain on caregivers is evident, as they reported higher rates of anxiety and depression compared to the general population. Financial challenges further compound caregiver burden, emphasizing the need for targeted support. By recognizing the multifaceted challenges faced by caregivers, healthcare systems can provide targeted resources and support to improve caregivers’ well-being and ultimately enhance the care they provide to their loved ones with MG. Moreover, these findings underline the need to improve treatment effectiveness for individuals with MG, not only to alleviate their own burden but also to reduce the burden posed on their caregivers.

## Data Availability

Due to privacy protections, the datasets generated during and analyzed during the current study are not publicly available. The data supporting our findings are available upon reasonable request through the corresponding author, Sarah Dewilde, via sd@she-consulting.be.

## Abbreviations

ALS: Amyotrophic Lateral Sclerosis
BSFC: Burden Scale for Family Caregivers
CB: Caregiver Burden
EQ-5D-5L: EuroQoL 5-Dimension 5-Level
GMP: Global Mental Health
GPH: Global Physical Health
HRQoL: Health related quality of life
MG: Myasthenia Gravis
MG-ADL: Myasthenia Gravis Activities of Daily Living
MID: Minimal important difference
MLD: Metachromatic leukodystrophy
PROMIS-GH 10: Patient-Reported Outcomes Measurement Information SystemSscale v1.2—Global Health 10 items
Q1, Q3: First quartile, Third quartile
SD: Standard deviation
VAS: Visual analogue scale
ZBI-22: Zarit Burden Interview – 22 items
